# Remarks on Mr. Evans' New Amalgam

**Published:** 1849-04

**Authors:** J. L. Levison


*Remarks on Mr. Evans' New Amalgam. To the Editor of the Lancet.*

Sir—In the last Lancet, there is a communication, entitled, "A New Amalgam for the Teeth," by Mr. Evans of Paris. The author says, "It is composed of chemically pure tin, prepared with much care, to insure its being free from any metallic substance, and combined with prepared cadmium, in small quantities, and mercury."

The remarks which I feel bound to make on this new compound cannot be regarded as invidious or personal, as some years since I called the attention of the profession to the injurious consequences arising from the use of amalgams and alloys for filling carious teeth. These compounds were shown to induce certain galvanic phenomena, and by rendering the saliva acidified, had a tendency ultimately to destroy the teeth. Every tyro in chemistry is aware that acids act chemically on the lime of teeth, which is the hardening and conservative portion of the enamel and dentine, and that if it is removed from time to time, however gradual the action may be, the ultimate tendency must be injurious, the injury being a certain consequence of exposing the pulp cavity. Sometimes, when the teeth are large and strong, and the general health good, the evil may not be experienced for some years, yet, in every case where amalgams are used, there is induced a sensitiveness in the teeth, under sudden changes of temperature, whether from cold or hot things taken in the mouth, or from the vicissitudes of the atmosphere. The amalgams hitherto in use are made with pure metals—e. g. gold or silver, or tin, and yet, whenever they are combined with crude mercury, they all oxidize more or less. In some mouths where there exists a strumous habit, or in dyspeptic complaints, this action takes place very rapidly. I cannot, therefore, consider that Mr. Evans' amalgam will be an exception to this rule. For he tells us, "In using it, (the new amalgam,) more or less mercury should be employed, as may be required to make it more or less plastic."

In some persons there is an idiosyncrasy to be soon affected with mercury, so that there are many cases on record that the use of amalgams often induces ptyalism. It cannot be the gold, or the silver, or the tin, to which these effects can be attributed, but only to the mercury combined with these pure metals.

I am induced to cite one fact related to me by one of the most eminent professors of the Queen's College, Birmingham. He said, "that he had an upper molar tooth stopped with amalgam, and in the lower jaw a molar stopped with gold. As these two teeth came in direct contact when the jaw was closed, he felt a perceptible galvanic action, and that if they could have been seen when such was the case, he had not the slightest doubt but that a spark would have been visible. Ultimately, the agony was so great in the tooth stopped with the amalgam, that he was obliged to have it extracted."

Trusting that you will insert this communication, from your usual impartiality in matters of scientific discussion, I am, sir, yours, respectfully,

J. L. LEVISON.

